# The Relationship between Primary Hyperparathyroidism and Thrombotic Events: Report of Three Cases and a Review of Potential Mechanisms

**Published:** 2018-07-01

**Authors:** Theocharis Koufakis, Vasiliki Antonopoulou, Maria Grammatiki, Spyridon N Karras, Ramzi Ajjan, Pantelis Zebekakis, Kalliopi Kotsa

**Affiliations:** 1 Division of Endocrinology and Metabolism, Diabetes Center, First Department of Internal Medicine, Medical School, Aristotle University of Thessaloniki, AHEPA Hospital, Thessaloniki, Greece; 2 Division of Cardiovascular and Diabetes Research, Leeds Institute for Cardiovascular and Metabolic Medicine, University of Leeds Ringgold Standard Institution, Leeds, UK

**Keywords:** Calcium, Primary hyperparathyroidism, Thrombosis, Stroke, Pulmonary embolism, Deep venous thrombosis

## Abstract

We have described three uncommon cases of patients who presented with clinical thrombotic events (stroke, pulmonary embolism and deep venous thrombosis) during the course of a hypercalcemia-induced hypercoagulable state. After thorough investigation, the diagnosis of primary hyperparathyroidism - due to a parathyroid adenoma - was established in all cases. The association between hypercalcemia and venous or arterial thrombosis has been previously described; however, relevant data are still insufficient. The existing evidence in the field was reviewed and the interesting underlying pathophysiologic mechanisms were also discussed. Further studies are required to shed more light on the unusual, still intriguing relationship between calcium and thrombosis.

## Introduction

 Hypercalcemia, a common electrolyte disorder in the clinical setting, is most frequently associated with primary hyperparathyroidism and malignancy^1^. Other, less common, causes include immobilization, drugs such as thiazide diuretics, granulomatous diseases (sarcoidosis, tuberculosis, lymphomas, among others) and augmented intake or absorption^2^. Primary hyperparathyroidism (PHPT) is characterized by the unregulated overproduction of parathyroid hormone (PTH) resulting in irregular homeostasis of calcium. The disease has been reported to have a prevalence of nearly 21 cases per 100.000 person-years, a mean age of diagnosis between 52 and 56 years, while it is considered significantly more common among females (female-to-male ratio of 3:1)^3^. With regards to its etiology, the majority of the cases (about 85%) are attributed to a single adenoma of the parathyroid glands. In the remaining 15%, the disease is associated with either multiple adenomas or hyperplasia, while only infrequently (less than 1%), a parathyroid carcinoma can be the cause of the disease^4^. 

The clinical spectrum of PHPT includes manifestations from multiple systems such as skeletal (osteitis fibrosa cystica), renal (kidney stones, polyuria), gastrointestinal (constipation, abdominal pain, peptic ulcers) and neurological (lethargy, confusion, stupor, and coma) ^5^. PHPT has been also linked with cardiovascular disease (CVD), as well as, increased morbidity and mortality^6^. Uncommon cardiovascular manifestations of PHPT include left ventricular hypertrophy, cardiac and vascular function abnormalities and increased carotid intima-medial thickness (IMT)^7^. Additionally, other CVD risk factors such as arterial hypertension, diabetes mellitus, increased Body Mass Index (BMI) and dyslipidemia have been described to correlate with PHPT^8^. 

The association between PHPT and thrombotic events, which has been previously reported in the literature, still little is known in terms of the pathophysiological mechanisms that connect these entities. It still remains uncertain whether the pathophysiological background of thrombosis in PHPT is related to prothrombotic factors acting indirectly (e.g. increased prevalence of risk factors for CVD), directly (causing disturbances of the hemostatic system) or even a combination of both. In this article, we have described three patients who presented with thrombotic events (stroke, pulmonary embolism and deep venous thrombosis, respectively) and after thorough investigation the diagnosis of PHPT - due to a parathyroid adenoma - was established in all cases. 

## Case 1 (Stroke)

A 63-year-old, female, left-handed patient presented to the Emergency Room with expressive aphasia for the last 5 hours. Her past medical history was unremarkable and she was not receiving any medications on a regular basis. Apart from aphasia, physical examination on presentation was normal. Blood samples were taken for initial laboratory evaluation and an urgent brain Computed Tomography (CT) scan was also performed. Her CT scan demonstrated an acute ischemic infarct at the left parietal lobe. At the same time, blood tests revealed severe hypercalcemia (14.1 mg/dl, reference range 8.8-10.5).

According to our hospital stroke protocol, patients are considered ineligible for thrombolysis, given that more than 4 hours have passed since the onset of symptoms. Her initial therapy included intravenous administration of saline, calcitonin and zolendronic acid. During the very first hours of her hospitalization, her neurological condition worsened, developing left hemiparesis and right- sided horizontal gaze paralysis (Foville’s syndrome). A second CT scan excluded intracranial hemorrhage and a Magnetic Resonance Imaging (MRI) brain scan after 48 hours showed another acute infarct at the anatomical area of the right pons. Unfortunately, due to technical reasons, we were not able to perform a CT angiography of the intracranial vessels. 

Further investigation revealed high serum PTH levels (11.1 pmol/l, 1.58-6.03), marginally low serum phosphorus levels (2.7 mg/dl, 2.7-4.5), vitamin D insufficiency [25(ΟΗ)D3 22 ng/ml, 30-100] and elevated urinary calcium excretion (325 mg/24h, 100-250) (Table 1). 

**Table 1:** Laboratory findings suggestive of the diagnosis of Primary Hyperparathyroidism in the described cases

**Laboratory parameter on** **presentation (Units, Reference** **Range)**	**Case 1** **(Stroke)**	**Case 2** **(PE)**	**Case 3** **(DVT)**
Calcium (mg/dl, 8.8-10.5)	14.1	15.5	15.1
PTH (pmol/l, 1.58-6.03)	11.1	72.1	79.3
Phosphorus (mg/dl, 2.7-4.5)	2.7	1.5	2.6
25(ΟΗ)D3 (ng/ml, 30-100)	22	18.2	7.1
24h urine calcium (mg, 100-250)	325	604	304

PTH: Parathyroid hormone, h: hours, PE: Pulmonary Embolism, DVT: Deep Venous Thrombosis

Imaging of the neck with CT, ultrasonography and scintigraphy of the parathyroid glands were suggestive for an adenoma of the right superior parathyroid gland. Renal ultrasound revealed bilateral nephrolithiasis. 

Potential causes of embolic stroke were excluded, given that electrocardiography (ECG), cardiac ultrasound, carotid artery triplex ultrasound and 24-hour holter monitoring demonstrated normal findings. Common risk factors for atherosclerosis, such as hypertension, smoking, dyslipidemia and diabetes were absent. Thrombophilia screening was negative. An electroencephalography (EEG) showed normal findings. Remarkably, her MRI did not bring to light any major atherosclerotic lesions of intracranial vessels.

Following the initial treatment, serum calcium levels returned to the normal range within 3 days accompanied by gradual improvement in symptoms. Ten days after admission, she was discharged with nifedipine, aspirin, normal serum calcium levels and only a mild paresis on her left hand. The patient was referred for surgical treatment of her hyperparathyroidism. Histological examination of the lesion was compatible with a benign parathyroid adenoma. Over repeated follow-up visits, she remained in an excellent physical condition without any evidence of neurological deficit within 2 months of intensive physiotherapy program. 

**Case 2 (Pulmonary Embolism)**

A 44-year-old, female patient presented to the Emergency Department with acute-onset, right-side chest pain, accompanied by fatigue and dizziness. Her medical history was remarkable only for recurrent episodes of renal colic. She denied smoking, frequent alcohol intake or therapy with any medication. Physical examination on presentation did not reveal any abnormal findings. Her blood pressure was 130/80mmHg, the heart pulse 80 /min, the respiratory rate 22/min, while the temperature was 35.9 °C. The oxygen saturation was 96% and the ECG demonstrated sinus rhythm. Laboratory evaluation revealed elevated d-dimers (4021 ng/ml, normal < 500) and serum calcium levels (15.04 mg/dl, 8.8-10.5). The aforementioned combination of clinical symptoms and laboratory findings raised the suspicion of Pulmonary Embolism (PE) and an urgent spiral CT angiography was ordered, which demonstrated a small filling defect in the hilum of the right lung. 

Low molecular weight heparin (LMWH) was administered for the treatment of PE, as well as intravenous hydration and calcitonin for the management of hypercalcemia. 

Further laboratory tests were ordered in order to identify the cause of hypercalcemia. PTH was measured excessively high (72.1 pmol/l, 1.58-6.03), while the 24-h urine calcium was respectively elevated (604 mg/24h, 100-250). Phosphorus (1.5mg/dl, 2.7-4.5) and 25(ΟΗ)D3 (18.2 ng/ml, 30-100) were found below the normal limits (Table 1). 

Ultrasonography was suggestive of an adenoma, sized 0.6 x 0.8 x 2.23 cm of the left inferior parathyroid gland. This finding was also confirmed by Tc99m Sestamibi imaging. Skeletal radiograph illustrated subperiosteal resorption of the phalanges, and renal ultrasound demonstrated a large coralloid renal stone in the left kidney. Bone densitometry (DXA scan) was indicative of osteoporosis (L1-L4 lumbar vertebras T-score -3.6, left neck femur T-score -2.8, right neck femur -3.8).

The assessment for blood coagulation abnormalities revealed high homocysteine levels (45.2 μmol/lt, normal <15) and homozygosity for Methylene tetrahydrofolate reductase (MTHFR) C677T mutation. No signs of DVT or findings suggestive of malignancy were found in imaging. 

After recovering from PE, the patient was referred for parathyroidectomy, and histological examination of the lesion was compatible with parathyroid adenoma. Following surgery, PTH and calcium levels returned to normal and the patient remained in good physical condition during her follow-up over a period of 6 months.

**Case 3 (Deep Venous Thrombosis)**

A 58-year-old female was referred by her General Practitioner to the Emergency Department due to lower right leg edema for 3 days. She had a history of Hashimoto thyroiditis and generalized arthralgia under investigation. She was on levothyroxine 125 mcg once daily. On presentation, the patient had a low-grade fever (37.5 ^o^ C), but was hemodynamically stable. Physical examination revealed tenderness, erythema, elevated temperature and edema in her right calf, while Homans sign (pain on dorsiflexion of the foot) was positive. Initial laboratory evaluation demonstrated abnormal d-dimers (3018 ng/ml, normal < 500) and serum calcium levels (15.1 mg/dl, 8.8-10.5). Triplex venous ultrasonography of the lower limbs was suggestive for extended DVT in the right leg. The patient was treated with LMWH, intravenous administration of saline, calcitonin and zolendronic acid. Additional investigation of hypercalcemia brought to light findings compatible with the diagnosis of PHPT [PTH 79.3 pmol/l, 24h calcium urine excretion 304 mg/24h, 25(ΟΗ)D3 7.1 ng/ml and phosphorus 2.6 mg/dl] (Table 1). Parathyroid sestamibi scintigraphy was indicative for an adenoma in the left lower parathyroid gland. CT scans of the thorax and abdomen, as well as mammography were negative for malignancy. The combination of long-term arthralgia and DVT raised the suspicion of an underlying autoimmune disorder. Multiple autoantibodies, including antiphospholipid, anti-cyclic citrullinated peptide (anti-CCP), anti-neutrophil cytoplasmic (ANCAs), antinuclear (ANA) and anti-double stranded DNA (anti-dsDNA) were measured, but were all negative. In the end, a lip biopsy confirmed the diagnosis of primary Sjogren’s Syndrome (SS).

The patient was subjected to parathyroidectomy, and histological examination of the removed lesion revealed a benign parathyroid adenoma. After surgery, both calcium and PTH levels returned to normal. She remains under the care of the rheumatology team for monitoring recently diagnosed SS.

## Discussion

 Back in 1973, Hilgard, studying a rat model, supported that acute hypercalcemia induces a hypercoagulable state^9^. Motivated by these findings, he suggested that hypercalcemia may also favor thrombus formation in vivo. However, to the best of our knowledge, recent clinical data supporting this view remain scarce.

**Figure 1:**
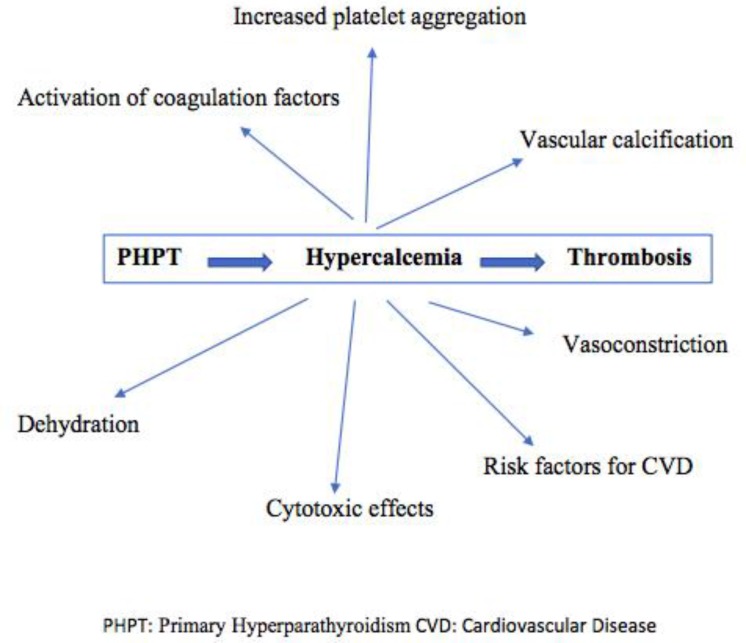
Potential mechanisms linking Primary Hyperparathyroidism and hypercalcemia with thrombosis

In the early 1970’s, Bostrom and Alveryd studied 170 patients with hypercalcemia, of whom 9 were diagnosed with stroke^10^. The same investigators randomly evaluated 2268 patients for hypercalcemia and found 12 with hyperparathyroidism, of whom 3 had lately been diagnosed with stroke. In the mid 1980’s, Gorelick and Kaplan reported 6 patients with hypercalcemia among a total number of 502 stroke patients ^11^. All of them had elevated PTH levels, but parathyroid adenoma and hyperplasia were detected in only 2 cases. Angiography was performed in 3 cases, demonstrating distal branch artery occlusion in 2 patients and distal branch narrowing, probably attributed to vasoconstriction, in the other case. Given the relatively low frequency of hyperparathyroidism in individuals sustaining a thrombotic event, it is likely that other contributory factors are required, and hypercalcaemia simply “facilitates” thrombotic vascular occlusion through various mechanisms (Figure 1) further discussed below.

The pathophysiologic background that connects calcium and thrombosis is not completely understood, however, several potential mechanisms have been proposed. It is believed that calcium triggers vascular smooth muscle leading to vasoconstriction ^12^^,^^13^ activates several factors of the clotting system and also boosts platelet aggregation^14^. Additionally, renal mechanisms for the reabsorption of sodium and water are disturbed by the hypercalcemic state, and non-compensated polyuria due to nausea and anorexia can lead to dehydration and hypercoagulable state^3^. Furthermore, elevated calcium concentrations can be potentially responsible for cytotoxic effects that stimulate cell death and result in thrombosis^15^.

A case-control study of 23 patients with PHPT showed that platelet count, FVII, FX activities, and D-Dimer levels were significantly increased in patients compared to controls^16^. In a later study, tissue plasminogen activator inhibitor-1 (PAI-1) and tissue plasminogen activator (t-PA) were also found to be increased, while the tissue factor pathway inhibitor (TFPI) was decreased in patients with PHPT compared to healthy controls ^17^. Elevated PAI-1 has been also reported in a cohort study of patients with PHPT and was found to correlate with PTH levels ^18^.

The presence of other mechanisms that act independently of hypercalcemia and promote atherosclerosis in patients with PHPT should also be considered. For example, recent data indicate that raised PTH levels are linked with high risk of cardiovascular disease in patients with chronic kidney failure even in the absence of elevated calcium and phosphate levels^19^. It seems that the key point which connects PTH and atherosclerosis in these cases is the vascular and valvular calcification, while reduction of PTH levels with cinacalcet has been proved to be protective against cardiovascular disorders^20^. The possibility that PHPT may synergically promote thrombosis when interacting with other prothrombotic factors such as mutations (as in case 2) or autoimmune diseases (as in case 3) cannot be excluded (Table 2). 

**Table 2:** Additional Risk Factors for thrombosis in the described cases

**Risk Factor**	**Case 1** **(Stroke)**	**Case 2 (PE)**	**Case 3 (DVT)**
Smoking	No	No	No
Arterial Hypertension	No	No	No
Diabetes Mellitus	No	No	No
BMI (kg/m^2^)	23	25.5	25
Genetic background	No	MTHFR C677T homozygosity	No
Autoimmunity	Νο	Νο	Hashimoto thyroiditis / Sjogren’s Syndrome

PE: Pulmonary Embolism, DVT: Deep Venous Thrombosis, BMI: Body Mass Index, MTHFR: Methylene tetrahydrofolate reductase

## CONCLUSION

 In conclusion, even being a rare cause of arterial and venous thrombosis, hypercalcemia should be always considered in the clinical assessment of patients with thrombosis, and calcium levels should be routinely requested in these cases. Furthermore, relevant clinical data connecting hypercalcemia and predisposition to thrombosis is still insufficient. Further studies are required in order to shed more light on the unusual, still intriguing relationship between calcium and thrombosis.
